# PfeIK1, a eukaryotic initiation factor 2α kinase of the human malaria parasite *Plasmodium falciparum*, regulates stress-response to amino-acid starvation

**DOI:** 10.1186/1475-2875-8-99

**Published:** 2009-05-12

**Authors:** Clare Fennell, Shalon Babbitt, Ilaria Russo, Jonathan Wilkes, Lisa Ranford-Cartwright, Daniel E Goldberg, Christian Doerig

**Affiliations:** 1 INSERM U609, Wellcome Centre for Molecular Parasitology, Biomedical Research Centre University of Glasgow, 120 University Place, Glasgow, G12 8TA, UK; 2 Institute of Immunology and Infection Reasearch, School of Biological Sciences, University of Edinburgh, West Mains Road, Edinburgh, EH9 3JT, UK; 3 Departments of Medicine and Molecular Microbiology, Howard Hughes Medical Institute, Washington University Box 8230, 660 S. Euclid Ave, St. Louis, MO 63110, USA; 4 Wellcome Centre for Molecular Parasitology, University of Glasgow, 120 University Place, Glasgow G12 8TA, UK; 5 Division of Infection and Immunity, Faculty of Biomedical and Life Sciences, University of Glasgow, 120 University Place, Glasgow G12 8TA, UK; 6 INSERM U609, Global Health Institute, Ecole Polytechnique Fédérale de Lausanne (EPFL), Station 19, CH-1015 Lausanne, Switzerland

## Abstract

**Background:**

Post-transcriptional control of gene expression is suspected to play an important role in malaria parasites. In yeast and metazoans, part of the stress response is mediated through phosphorylation of eukaryotic translation initiation factor 2α (eIF2α), which results in the selective translation of mRNAs encoding stress-response proteins.

**Methods:**

The impact of starvation on the phosphorylation state of PfeIF2α was examined. Bioinformatic methods were used to identify plasmodial eIF2α kinases. The activity of one of these, PfeIK1, was investigated using recombinant protein with non-physiological substrates and recombinant PfeIF2α. Reverse genetic techniques were used to disrupt the *pfeik1 *gene.

**Results:**

The data demonstrate that the *Plasmodium falciparum *eIF2α orthologue is phosphorylated in response to starvation, and provide bioinformatic evidence for the presence of three eIF2α kinases in *P. falciparum*, only one of which (PfPK4) had been described previously. Evidence is provided that one of the novel eIF2α kinases, PfeIK1, is able to phosphorylate the *P. falciparum *eIF2α orthologue *in vitro*. PfeIK1 is not required for asexual or sexual development of the parasite, as shown by the ability of *pfeik1*^- ^parasites to develop into sporozoites. However, eIF2α phosphorylation in response to starvation is abolished in *pfeik1*^- ^asexual parasites

**Conclusion:**

This study strongly suggests that a mechanism for versatile regulation of translation by several kinases with a similar catalytic domain but distinct regulatory domains, is conserved in *P. falciparum*.

## Background

Human malaria is caused by infection with intracellular protozoan parasites of the genus *Plasmodium *that are transmitted by *Anopheles *mosquitoes. Of four species that infect humans, *Plasmodium falciparum *is responsible for the most virulent form of the disease. The transition from one stage of the life cycle to the next must be tightly regulated, to ensure proliferation and differentiation occur when and where appropriate; this is undoubtedly linked to differential gene expression. Analysis of the *P. falciparum *transcriptome during the erythrocytic asexual cycle reveals an ordered cascade of gene expression [1], and the various developmental stages display distinct transcriptomes; how this is orchestrated remains obscure. Initial investigation of the *P. falciparum *genome revealed a paucity of transcriptional regulators [2], although this picture has recently been challenged by the recent identification of the ApiAP2 transcription factor family [3]. There is nevertheless a large body of evidence suggesting that post-transcriptional control is an important means of gene regulation in *P. falciparum*. Examples include the relatively small number of identifiable transcription-associated proteins, abundance of CCCH-type zinc finger proteins commonly involved in modulating mRNA decay and translation rates [2] and translational repression during gametocytogenesis [4-6].

In mammalian cells, regulation of gene expression is a key mechanism in the mediation of stress responses, which may be achieved by influencing transcription or translation. The Stress Activated Protein kinases (SAPKs), specifically JNKs and p38 kinases, are subfamilies of mitogen activated protein kinases (MAPK) that are expressed in most eukaryotic cells, and respond to a variety of stress conditions [7]. Although the parasite kinome includes two MAPK homologues, none of these are members of the SAPK subfamily [8-10]. In contrast, the *P. falciparum *kinome contains a phylogenetic cluster of three kinases with homology to eukaryotic Initiation Factor 2α (eIF2α kinases, which in other organisms regulate translation in response to stress [10]. Interestingly, the related apicomplexan parasite *Toxoplasma gondii *has been shown to differentiate from tachyzoites to bradyzoites on exposure to a number of cellular stresses, concomitant with an increase in phosphorylation of TgeIF2α, indicating a possible role for this mechanism in parasite differentiation [11].

Phosphorylation of eukaryotic initiation factor 2α at residue Ser51 in response to stress is a well-characterized mechanism of post-transcriptional control that regulates initiation of translation [12-17]. In mammalian cells this phosphorylation event is mediated by four distinct protein kinases, called the eIF2α kinases: general control non-derepressible-2 (GCN2), haem-regulated inhibitor kinase (HRI), RNA-dependent protein kinase (PKR), and PKR-like endoplasmic reticulum kinase (PERK). These enzymes contain a similar catalytic domain allowing them to phosphorylate the same substrate, but have different accessory domains that regulate kinase activation in response to different signals. In GCN2 the functional kinase domain is followed by a histidyl-tRNA synthetase (HisRS)-like domain [18], which is the major motif for sensing amino acid starvation and triggering kinase activation; PERK has a transmembrane domain allowing it to reside in the endoplasmic reticulum membrane; the N-terminal domain can protrude into the lumen of the ER to sense unfolded proteins, while the catalytic domain extends into the cytoplasm where its substrate and effector mechanism lie; human PKR contains an RNA binding domain and responds to viral infection; and HRI contains haem binding sites to modulate translation of globin chains according to the availability of haem. In this way the eIF2α kinases can integrate diverse stress signals into a common pathway [12-14,19].

Translation initiation requires the assembly of the 80S ribosome on the mRNA, which is mediated by proteins known as eukaryotic initiation factors (eIFs). Formation of the 43S pre-initiation complex depends on binding of the ternary complex that consists of the heterotrimeric G-protein eIF2 (α, β and γ subunits), methionyl-initiator tRNA (met-tRNAi) and GTP [13]. Initiation of translation and release of the initiation factors involves hydrolysis of GTP to GDP, which leaves an inactive eIF2-GDP complex. Before further rounds of translation initiation can occur eIF2 must be reactivated by exchange of GDP for GTP [13]. The presence of a phosphate group on the α subunit of eIF2 inhibits recycling of inactive eIF2-GDP to active eIF2-GTP by limiting the activity of the guanine nucleotide exchange factor, eIF2B [20]. The consequence of activity of the eIF2α kinases therefore is global translation repression, since initiation complexes cannot form. In spite of the generalized reduction in translation, selected mRNAs are translated, whose products shapes the subsequent stress response. Reduced translation conserves energy and nutrients, allowing time for the cell to adapt appropriately to the stress conditions. This mechanism is conserved in the vast majority of eukaryotes. One notable exception is the Microsporidium *Encephalitozoon cuniculi*, whose kinome does not include eIF2α kinases (or other stress-response kinases), a probable adaptation to its parasitic lifestyle [21]. It is, therefore, of interest to investigate the extent to which malaria parasites may rely on eIF2α phosphorylation for stress-response and/or life cycle progression.

A cluster of three sequences that includes PfPK4, a protein kinase that was previously described as a putative eIF2α kinase [22], was identified in the *P. falciparum *kinome on the basis of catalytic domain similarity [10,23]. Here, evidence is provided that the *P. falciparum *eIF2α orthologue is phosphorylated in response to amino acid starvation. Bioinformatics analysis reveals that *P. falciparum *encodes three eIF2α kinases, one of which, *Plasmodiumfalciparum *eukaryotic Initiation Factor Kinase-1 (PfeIK1), is indeed be able to phosphorylate *P. falciparum *eIF2α *in vitro*. Reverse genetics experiments show that inactivation of the *pfeik1 *gene does not affect asexual growth, gametocytogenesis or further sexual development, since *pfeik1*^- ^sporozoites can be formed in the mosquito vector; in contrast, *pfeik1*^- ^parasites are unable to phosphorylate eIF2α in response to amino-acid starvation.

## Methods

### Bioinformatic analysis

BLASTP analysis was used to identify the closest human and *Plasmodium berghei *orthologues of the PfeIF2α kinases. Catalytic domains of the putative PfeIF2α kinases as defined by the alignment of *P. falciparum *kinases [10] were aligned with the four human eIF2α kinases and other *P. falciparum *and human sequences that were selected to represent all kinase subfamilies. The sequences were aligned using the HMMER package against a profile generated from our previous kinome analysis [10]. After removal of gaps and positions with a low quality of alignment, alternate phylogenies generated with the neighbour joining method were visualized using NeighbourNet implemented on SplitsTree version 4 [24].

BLASTP searches of PlasmoDB using metazoan eIF2α sequences as queries identified PF07_0117 as the *P. falciparum *homologue of eIF2α, which was then confirmed by reciprocal analysis. Alignment of these sequences was performed using ClustalW.

### Molecular cloning

#### PfeIK1

A 1278 bp fragment encoding the catalytic domain of PfeIK1 (PF14_0423) was amplified from a *P. falciparum *cDNA library using the *Phusion *polymerase (Finnzymes), using the following primers: forward, GGGGGGATCCATGGGGAAAAAAAAACATGG, reverse GGGGGTCGACCGTAAAAAGTACACTTTCGTG. The primers contained *Bam*HI and *Sal*I restriction sites, respectively (underlined). The *Taq *polymerase (Takara) was used to add adenine tails to enable cloning of the product into the pGEM-T Easy vector (Promega) for sequencing. The correct sequence was removed by digestion with *Bam*HI and *Sal*I and inserted into the expression vector pGEX-4T3 (Pharmacia). A catalytically inactive mutant was obtained by site directed mutagenesis of Lys^458 ^to Met using the overlap extension PCR method [25] (forward: CTTATGCATTAATGATTATAAG, reverse: CTTATAATCATTAATGCATAAG).

#### PfeIF2α

Oligonucleotide primers were designed to amplify the complete coding sequence of PfeIF2α (PF17_0117) by PCR from a cDNA library of the *P. falciparum *clone 3D7, using the *Phusion *polymerase (Finnzymes). The primers used were as follows: forward, GGGGGGATCCATGACTGAAATGCGAGTAAAAGC and reverse, GGGGGTCGACTTAATCTTCCTCCTCCTCGTC (restriction sites are underlined). *Taq *polymerase (Takara) was used to add adenine tails to enable cloning of the 990 bp product into the pGEM-T Easy vector (Promega) for amplification and sequencing. The correct sequence was removed by digestion with *Bam*HI and *Sal*I and inserted into the expression vector pGEX-4T3 (Pharmacia). A mutant of PfeIF2α designed to be refractory to phosphorylation was obtained by site directed mutagenesis (Ser^59 ^– Ala) using the overlap extension PCR technique [25], (primers: forward, CTTATGCATTAATGATTATAAG, reverse, CTTATAATCATTAATGCATAAG).

All inserts were verified by DNA sequencing (The Sequencing Service, Dundee, UK) prior to expression of recombinant proteins or transfection of *P. falciparum*.

### Recombinant protein expression

Expression of recombinant GST fusion proteins was induced in *E. coli *(strain BL21, codon plus) with 0.25 mM Isopropyl Thiogalactoside (IPTG). After induction, bacteria were grown at 16°C overnight and the resulting bacterial pellets were stored at -20°C until use. All subsequent work was done on ice, centrifugation steps at 4°C. Protein extraction was performed by digestion of bacterial pellets for 5 min. with lysozyme (Sigma), followed by 10 min. in lysis buffer (1 × PBS, 2 mM ethylenediaminetetraacetic acid (EDTA), 1 mM dithiothreitol (DTT), 0.5% Triton ×100, 1 mM Phenyl Methyl Sulphonate (PMSF), Benzamidine Hydrochloride Hydrate (BHH), 1× complete cocktail protease inhibitors (Roche)). Bacterial lysates were sonicated at 20% amplitude (Bioblock Scientific, Vibracell 72405), 5 × 15 sec. pulses/15 sec. rest, and cleared by centrifugation 13000 g, 15 min. GST-fusion proteins were purified by incubation of cleared lysates on glutathione agarose beads (Sigma) for 2 hours, followed by four washes with lysis buffer and eluted for 20 min. in elution buffer (Tris 40 mM, pH8.7, 75 mM NaCl, 15 mM reduced glutathione). Protein concentration was monitored using the Bradford assay (Biorad reagent). Kinase assays were carried out immediately after purification.

### Kinase assay

Kinase reactions (30 μl) were carried out in a standard kinase buffer containing 20 mM Tris-HCl, pH 7.5, 20 mM MgCl_2_, 2 mM MnCl_2_, phosphatase inhibitors; 10 mM NaF, 10 mM β-glycerophosphate, 10 μM ATP and 0.1 MBq [γ-^32^P] ATP, using 2 μg recombinant kinase, and 10 μg non-physiological substrate (α-casein, β-casein), or recombinant GST-PfeIF2α. Reactions were allowed to proceed for 30 min. at 30°C and stopped by addition of reducing Laemmli buffer, 3 minutes, 100°C. Samples were separated by SDS-PAGE and phosphorylation of kinase substrates assessed by autoradiography of the dried gels.

### *Plasmodium falciparum *genetic manipulation

A gene disruption plasmid was produced for PF14_0423 in the plasmid pCAM-BSD [26] that contains the gene conferring resistance to blasticidin. The oligonucleotide pair GGGGGGATCCGTAATGAAAGTAAAAAATAAG/GGGGCGCCGGCGAGGTGAAATATAATGAATTGTTCC, containing *Bam*HI and *NotI *sites (underlined) was used to amplify a 789 bp fragment for insertion to pCAM-BSD. Ring stage parasites were electroporated with 50 – 100 μg plasmid DNA, as previously described [26]. Blasticidin (Calbiochem) was added to a final concentration of 2.5 μg/ml 48 hours after transfection to select for transformed parasites. Resistant parasites appeared after 3–4 weeks and were maintained under selection. After verification by PCR that *pfeik1*^- ^parasites were present, the population was cloned by limiting dilution in 96 well plates (0.25/0.5/1.0 parasite per well). Genotypic analysis enabled selection of independent *pfeik1*^- ^clones for further phenotypic analysis.

### Parasite culture and mosquito infection

*Plasmodium falciparum *clone 3D7 was cultured as previously described [27]. In brief, asexual cultures were maintained in complete RPMI at a haematocrit of 5%, between 0.5% and 10% parasitaemia. Asexual growth cycle was analyzed by flow cytometric assessment of DNA content as previously described [28]. Gametocytogenesis was induced as described previously [29]; briefly, gametocyte cultures were set up at 0.5–0.7% parasitaemia in 6% haematocrit (using human blood not more than 7 days after the bleed), in an initial volume of 15 ml in 75 cm^2 ^flasks. Cultures were maintained for 4–5 days until 8–10% parasitaemia was reached and parasites appeared stressed, after which the volume was increased to 25 ml. For each clone a mixture of day 14 and day 17 gametocyte cultures were fed to *Anopheles gambiae*, through membrane feeders as described [29]. Female mosquitoes were dissected 10 days post-infection and midguts examined by light microscopy for presence of oocysts. Sporozoite invasion of salivary glands was assessed by removal of salivary glands 16 days post-infection and examination by light microscopy. DNA was extracted from oocyst-positive midguts using previously published methods [30]. Fisher's exact test was used to compare infection prevalence between oocyst and sporozoite stages, where appropriate.

### Preparation of parasite pellets

Parasite pellets were obtained by saponin lysis: erythrocytes were centrifuged at 1300 g for 2 min. at room temperature, washed in an equal volume of Phosphate Buffered Saline (PBS), pH 7.5, and centrifuged at 1300 g for 2 min. at 4°C. Erythrocytes were lysed on ice by resuspension and repeated pipetting in 0.15% saponin in PBS. The PBS volume was then increased and parasites recovered by centrifugation at 5500 g for 5 min., at 4°C. After two washes in PBS, the parasite pellets were stored at -80°C.

### *Plasmodium falciparum *amino acid starvation assay

*Plasmodium falciparum *3D7 parasites and clonal lines of *pfeik1*^- ^and *pfeik2*^- ^parasites were synchronized to the late ring stage, cultured in complete RPMI at 2% haematocrit, and grown to approximately 8 – 10% parasitaemia. The parasites were washed two times in 1× PBS, equally partitioned and washed with either complete RPMI or RPMI medium lacking amino acids, after which, the parasites were re-plated in their respective medium. The plates were incubated at 37°C with 5% CO_2 _for 5 hours. After 5 hours, one culture maintained in amino acid free medium was supplemented with complete RPMI, and re-incubated at 37°C for an additional 45 minutes. Post-incubation, the parasites were isolated by tetanolysin (List Biological) treatment, washed with 1× PBS buffer containing Complete™ protease inhibitor cocktail (Roche), 2 mM NaF, and 2 mM Na_3_VO_4_. Samples were resuspended in 2× SDS-Laemmli buffer. Parasite proteins were resolved by SDS-PAGE and transferred to nitrocellulose for immunoblotting.

### Antibodies and immunoblotting

Rabbit anti-phosphorylated eIF2α (Ser 51) was purchased from Cell Signaling Technology (Danvers, MA). Rat anti-BiP was acquired from the Malaria Research and Reference Reagent Resource Center (ATCC, Manassas, VA). Secondary antibodies used were conjugated with horseradish peroxidase (HRP). For immunoblotting, nitrocellulose membranes were blocked with 5% BSA in TBS-0.1% Tween 20 (TBST) for 1 hour at room temperature. Rabbit anti-phosphorylated eIF2α (Ser 51) was diluted 1:1000 in TBST. Rat anti-BiP was diluted 1:10,000 in TBST. Respective secondary antibodies were diluted 1:20,000. Bound antibodies were detected with Western Lightning™ Chemiluminescence reagent (Perkin Elmer).

### Southern blotting

To obtain genomic DNA, parasite pellets were resuspended in PBS and treated with 150 μg/ml proteinase K and 2% SDS at 55°C for 4 hours. The DNA was extracted using phenol/chloroform/isoamyl alcohol (25:24:1), and precipitated in ethanol with 0.3 M sodium acetate at -20°C. Restriction digests were carried out with *Hind*III. Probes were labelled with alkaline phosphatase using the Gene Images AlkPhos Direct Labelling kit (Amersham).

## Results and Discussion

### Stress-dependent phosphorylation of the *P. falciparum *eIF2α orthologue

**Figure 1:**
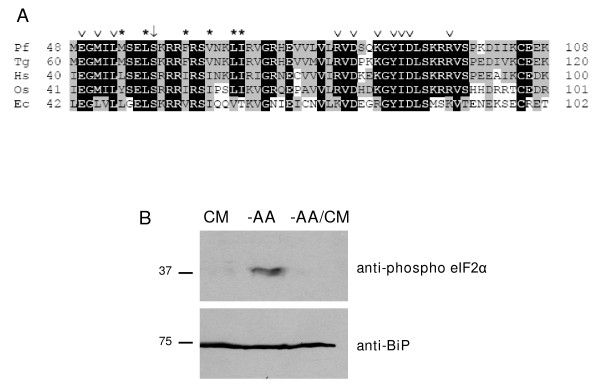
**The *P. falciparum *eIF2α orthologue is phosphorylated in response to amino-acid starvation**. A: Alignment of PfeIF2α with orthologous sequences from *T. gondii *(Tg), human (Hs), rice (Os) and *E. cuniculi *(Ec). Sequences surrounding the conserved regulatory serine, (*P. falciparum *numbering: M48 – K108) are shown. Residues that are identical in all sequences are highlighted in black, residues that are identical or similar are marked in grey. The arrow indicates the serine that is the target of eIF2α kinases. Open arrow heads (∨) indicate residues involved in contacting the kinase domain, asterisks (*) indicate conserved residues that protect the phosphorylation site from the activity of other kinases. B. Western blot analysis of PfeIF2α phosphorylation. A 3D7 parasite culture synchronized to the late ring stage was equally partitioned into individual cultures. Growth of the parasites was continued up to 5 hours at 37°C in either complete RPMI medium (CM) or in RPMI lacking amino acids (-AA). CM was added back to one amino acid-deprived culture, and re-incubated for an additional 45 minutes. Total lysates from the parasites were prepared for SDS-PAGE, followed by immunoblotting with antibodies against phosphorylated eIF2α (anti-phospho eIF2α) and the endoplasmic reticulum (ER) marker, BiP (anti-BiP), which served as the loading control.

BLASTP searches of PlasmoDB using metazoan eIF2α sequences were used to identify PF07_0117 as the *P. falciparum *orthologue, which was confirmed by reciprocal analysis. The alignment of *P. falciparum *eIF2α with sequences from *T. gondii*, human, rice and *E. cuniculi *is shown in Figure 1A. Overall, the *P. falciparum *sequence shares ~70% identity with *T. gondii *eIF2α and ~50%, ~40% and ~28% with the orthologues in humans, rice and *E. cuniculi*, respectively. Importantly, the serine that is targeted for phosphorylation is conserved in all species. Furthermore, eIF2α contacts the kinase through a large number of residues that interact with the surface of the kinase domain. These residues are also conserved in most species, as are residues that protect the regulatory serine from the activity of other kinases [31] (Figure 1A); interestingly, several of these are not conserved in the *E. cuniculi *orthologue, which is consistent with the absence of eIF2α kinases in this organism [21].

The presence of the target serine residues, and of residues which in other species are involved in interaction with eIF2α kinases, suggests that PfeIF2α may be regulated by phosphorylation under stress conditions. To test this hypothesis, cultured intraerythrocytic parasites were starved of amino acids, and the phosphorylation status of PfeIF2α was monitored by western blot using an antibody that specifically recognizes the phosphorylated form (Ser51) of human eIF2α, reasoning that the high level of sequence conservation between the human and plasmodial sequences would allow cross-reaction of the antibody (Figure 1B). Indeed, the antibody recognized the expected 37-kDa band in parasite extract, and the intensity of the signal was considerably stronger in the lane containing extracts from parasites that had been stressed by amino-acid starvation than in extracts from unstressed parasites, despite equal quantities of the eIF2α factor (as quantitated with a non-phosphodependent antibody). Furthermore, this effect was removed by restoring the amino acids in the culture medium. This demonstrates that the *P. falciparum *equivalent residue of human eIF2α Ser51 is phosphorylated in response to starvation.

### Identification of eIF2α kinases in *P. falciparum*

**Figure 2:**
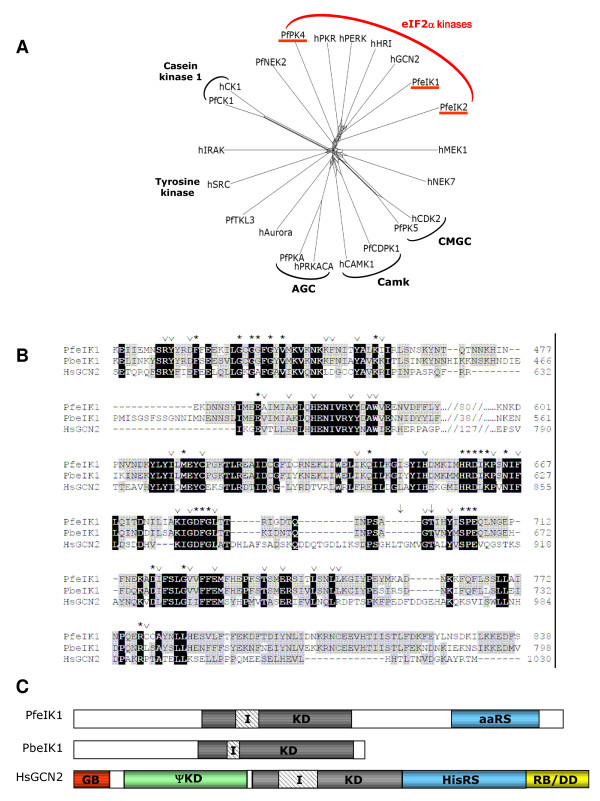
**Bioinformatic analyses of *P. falciparum *eIF2α kinases**. A: Phylogenetic tree showing clustering of PfeIF2α kinases with human eIF2α kinases. Sequences: PfeIK1: PF14_0114; PfeIK2: PFA0380w; PfPK4: PFF1370; PKR: GI:4506103; HRI: GI:6580979; PERK: GI:18203329; GCN2: GI:65287717; MEK1: GI:400274; IRAK1: GI:68800243; Aurora: GI:37926805; CDK2: GI:1942427; PfCK1: PF11_0377; PfNEK2: Pfe1290w; PfPK5: MAL13P1.279; PfPKA: PFI1685w; PFTKL3: PF13_0258; Pfb0815w: PfCDPK1; hCAMK1: GI:4502553; hPRKACA: GI:46909584; hCSNK1d: GI:20544145; hNEK7: GI:19424132; hSRC: GI:4885609. B. Alignment of the catalytic domains of PfeIK1, PbeIK1 and human GCN2. Identical residues in all three kinases are in black boxes, residues that are identical in two sequences of the three sequences, or that are similar are boxed in grey. The number of residues comprising the inserts between domains IV and V are marked between //-//. Asterisks (*) mark residues conserved among kinases in general, while open arrowheads (∨) indicate residues specifically conserved among eIF2α kinases. The downwards arrow marks the threonine residues that are targets for autophosphorylation in GCN2. PlasmoDB accession numbers: PfeIK1: PF14_0423, PbeIK1: PB000582.03.0 GenBank accession number: HsGCN2: GI:65287717. C: Schematic of the domain structures of PfeIK1, PbeIK1 and GCN2. Kinase domains (KD) are in grey, hatched regions represent the inserts (I) within the kinase domains and regions with no identified function are white. Additional characterized domains of GCN2 are as follows: red; N-terminal GCN1 binding domain (GB), green; pseudo-kinase domain (ΨKD), blue; histidyl-tRNA synthetase (HisRS), yellow; ribosome binding and dimerisation domain (RB/DD).

Bioinformatics approaches were then used to identify *P. falciparum *protein kinase(s) potentially responsible for this response. An analysis of the complete complement of *P. falciparum *protein kinases [10] identified a distinct phylogenetic cluster of three sequences, PF14_0423, PFA0380w and PFF1370, the latter of which (called PfPK4) had previously been characterized as an eIF2α kinase [22]. Reciprocal BLASTP analysis using the putative catalytic domains as queries confirmed the homology of these three genes with the eIF2α kinase family. A Hidden Markov Model (HMM) was used to generate an alignment of the three *P. falciparum *sequences with those of human eIF2α kinases; sequences of kinases from other families were included as outgroups. The resulting alignment was used to generate a phylogenetic tree (Figure 2A), which clearly shows that the three *P. falciparum *genes cluster with the eIF2α kinases, as opposed to other kinase families, confirming their relatedness to this family. Interestingly PfeIK1 (PF14_0423), on which the present study focuses, clusters most closely with GCN2, which is suggestive of a role in response to nutrient levels.

The PF14_0423 gene model proposed in PlasmoDB [32] predicts a single intron that falls close to the 5' end of the sequence so that the kinase domain is encoded entirely within the second exon. All the residues that are required for catalytic activity [33] are present in the kinase domain, suggesting the gene encodes an active enzyme. The sequence shares the feature of insertions within the catalytic domain with other eIF2α kinases [34] (Figures 2B and 2C). Three of the human eIF2α kinases have N-terminal extensions containing regulatory domains; the fourth, GCN2, has extensions on either side of the kinase domain (reviewed in [35]). As PfeIK1 has extensions on both sides of the catalytic domain, it is most similar to GCN2 not only in the sequence of its catalytic domain, as the phylogenetic tree (Figure 2A) demonstrates, but also in overall structure (Figure 1C). Furthermore, the C-terminal extension of PfeIK1 contains an "anti-codon binding" domain (Superfamily entry SSF52954) that may mediate binding to uncharged tRNAs, a function that is performed in GCN2 by the HisRS domain present in the C-terminal extension (Figure 1C) [18]. This adds weight to the possibility that PfeIK1 is involved in the response to amino acid starvation, like GCN2. The other functional domains present in the GCN2 extensions were not recognisable in PfeIK1.

### Kinase activity of recombinant PfeIK1

**Figure 3:**
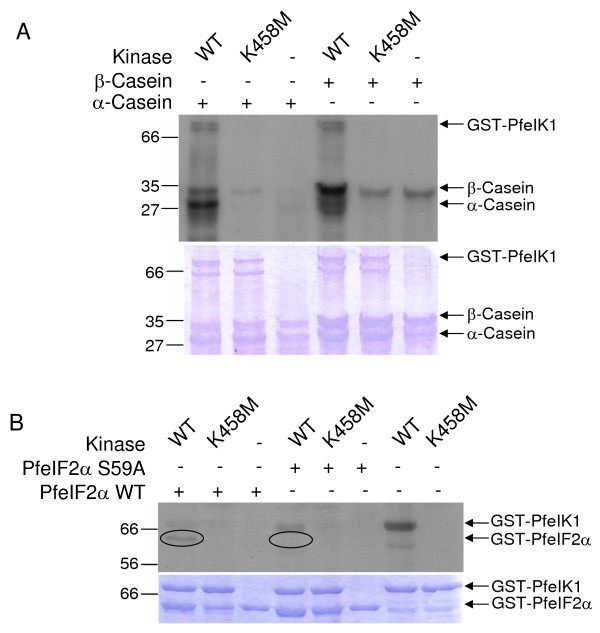
**Kinase activity of PfeIK1**. A: GST-PfeIK1 phosphorylates the exogenous substrates α-and β-casein. Kinase assays were performed using 10 μg α-casein (left 3 lanes) or β-casein (right 3 lanes), in the presence of 2 μg wild-type kinase catalytic domain (WT), catalytically inactive mutant (K458M) or no kinase (-). Upper panel: autoradiogram, lower panel: Coomassie blue-stained gel. B: GST-PfeIK1 autophosphorylates and can phosphorylate recombinant GST-PfeIF2α, but not the mutant GST-PfeIF2α-S59A. Kinase assays were performed using 2 μg wild-type PfeIK1 catalytic domain (WT), or catalytically inactive mutant (K458M), or no kinase (-), in the presence of 10 μg wild-type GST-PfeIF2α (left 3 lanes), targeted mutant GST-PfeIF2α-S59A (middle 3 lanes) or no substrate (right 2 lanes). The position of the substrate is highlighted by ovals.

In order to verify that the *pfeik1 *gene encodes a functional kinase, the catalytic domain was expressed as a GST fusion protein in *E. coli*. A recombinant protein of the expected size (76 kDa) was obtained and purified for use in kinase assays. The protein appeared as a doublet in most preparations, with both bands reacting with an anti-GST antibody. Kinase assays were performed with α- or β-casein as substrates, in the presence or absence of GST-PfeIK1 (Figure 3A). A weak signal was detectable with β-casein on the autoradiogram even in the absence of the kinase, indicating a low level of contaminating kinase activity in the substrate itself. This signal was much stronger in the presence of GST-PfeIK1, and a signal was also observed with α-casein, which was not labelled in the absence of the kinase. Furthermore, a signal at a size matching that of the upper band in the GST-PfeIK1 doublet was also seen, indicating possible autophosphorylation, an established property of at least some mammalian eIF2α kinases, including GCN2 [34,36-38]. GCN2 autophosphorylation occurs on two threonine residues in the activation loop [36], only one of which conserved in PfeIK1 (Figure 2B). Autophosphorylation was more clearly seen in the absence of any exogenous substrate (Figure 3B). The possible functional relevance of PfeIK1 autophosphorylation remains to be determined. Taken together, these data suggest that PfeIK1 possesses catalytic activity. To ensure that the signals were not due to co-purified activities from the bacterial extract, the assays were repeated using a catalytically inactive mutant (Lys458→Met) of GST-PfeIK1. These reactions yielded an identical pattern as the reaction containing no recombinant kinase (Figure 3A), confirming that the phosphorylation of the caseins is due to GST-PfeIFK1, and that the recombinant kinase can autophosphorylate.

In order to establish whether PfeIK1 is an eIF2α kinase as predicted, its activity was tested towards recombinant *P. falciparum *eIF2α expressed as a 64 kDa GST fusion. Figure 3B (left lane) shows that GST-PfeIK1 can phosphorylate wild-type GST-PfeIF2α. The signal appears very weak, which may be explained by the fact that the recombinant kinase contains only the catalytic domain and may not mimic the enzyme in a fully activated, physiological status. Indeed, an activation mechanism for GCN2 has been proposed [37], in which a conformational alteration of the so-called "hinge region" of the catalytic domain is induced by uncharged tRNA binding to the HisRS domain, which would favour productive binding of ATP to the active site. Such a positive effect of the regulatory domain would not be possible with GST-PfeIK1, since it contains only the catalytic domain.

Consistent with the hypothesis that PfeIK1 may regulate translation through PfeIF2α phosphorylation, mutation of the predicted target for phosphorylation in the substrate (Ser59→Ala) prevents labelling with the recombinant enzyme (Figure 3B).

### Generation of *pfeik1*^- ^clones

**Figure 4:**
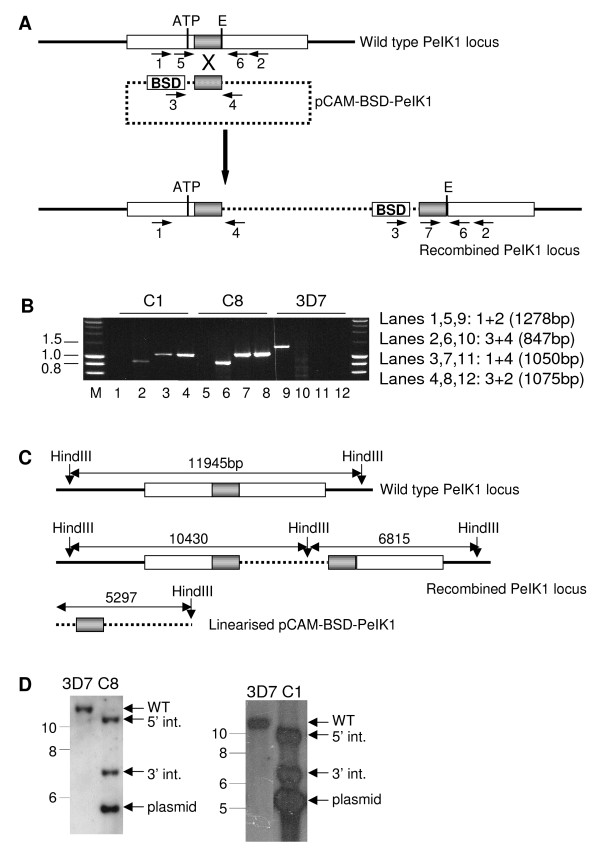
**Disruption of the *pfeik1 *gene**. A. Strategy for gene disruption. The transfection plasmid contains a PCR fragment spanning positions 1467–2255 of the entire 4.8 kb *pfeik1 *coding sequence (as predicted on PlasmoDB). The fragment excludes two regions essential for catalytic activity, labelled 'ATP' (a glycine-rich region required for orientation of ATP) and 'E' (a glutamate residue required for structural stability of the enzyme). The positions of primers used for genotyping clones, and for nested PCR to genotype oocsyts are indicated by numbered arrows. B: PCR analysis. Genomic DNA isolated from *pfeik1*^- ^clones C1 and C8, and from 3D7 wild-type parasites, was subjected to PCR using the indicated primers (see Figure 4A for primer locations). Lanes 1, 5, 9: primers 1 + 2 (diagnostic for the wild-type locus); lanes 2, 6, 10: primers 3 + 4 (diagnostic for the pCAM-BSD-PfeIK1 plasmid); lanes 3, 7, 11: primers 1 + 4 (diagnostic for 5' integration boundary); lanes 4,8,12: primers 3 + 2 (diagnostic for 3' integration boundary). M = co-migrating markers. C: Schematic of expected sizes on Southern blot analysis of wild-type 3D7 parasites and *pfeik1*^- ^parasites. D: Southern blot analysis of the *pfeik1 *locus in wild-type 3D7 and *pfeik1*^- ^clones C1 and C8. Genomic DNA was digested with *Hind*III, transferred to a Hybond membrane and probed with the *pfeik1 *fragment that was used as the insert in the pCAM-BSD-PfeIK1 plasmid. Positions of the bands corresponding to the wild-type locus (WT), 5' integration (5' int.), 3' integration (3' int.) and linearized plasmid (plasmid) are shown on the right. Sizes of co-migrating markers are indicated on the left.

Microarray data available in PlasmoDB [1,39] indicate that *pfeik1 *is expressed in asexual parasites; it can be hypothesized that the kinase plays a role in the parasite's stress response, and may therefore (i) not be essential for the asexual cycle, and (ii) be involved in regulation of gametocytogenesis, similar to the function of a eIF2α kinase in *T. gondii *stage transition from tachyzoite to bradyzoite. *P. falciparum *clones that do not express PfeIK1 were generated to test these hypotheses. The strategy used to disrupt expression of the kinase relies on single cross-over homologous recombination, and has been used successfully to knock-out other *P. falciparum *protein kinase genes [40,41]. Briefly, a plasmid based on the pCAM-BSD vector [26] containing a cassette conferring resistance to blasticidin and an insert comprising the central region of the PfeIK1 catalytic domain, was transferred by electroporation into asexual parasites of the 3D7 clone. Homologous recombination is expected to generate a pseudo-diploid locus in which neither of the two truncated copies encodes a functional kinase: the 5' copy lacks an essential glutamate residue in subdomain VIII and all downstream sequence including the 3'UTR; the 3' copy lacks the both the promoter region and the essential ATP orientation motif in subdomain I (Figure 4A).

**Figure 5:**
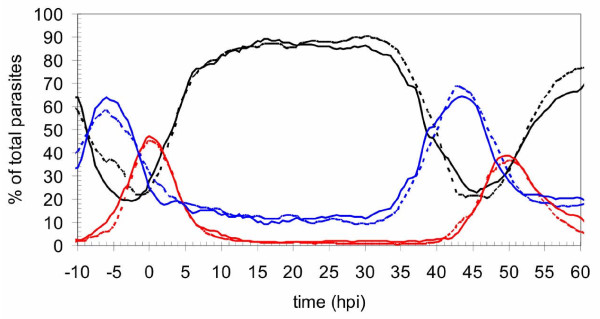
**Disruption of the *pfeik1 *gene does not affect asexual growth rate**. Representative cycles of *pfeik1*^-^parasites and the parental 3D7 strain (dashed). Cycle points were semi-automatically collected fixed and stored at 4°C every 30 min over ~4 days. After permeabilization and RNAse treatment, the DNA content was analyzed by flow cytometry as previously described [42]. Mature schizonts (~16–32 N), red line; S-phase (~2–8 N), blue line; G1-phase (1N), black line. Percentage values as a function of time are shown; hpi: hours post-infection, referring to the mature schizont maxima as zero.

Blasticidin-resistant parasite populations were obtained and shown by PCR analysis to contain parasites whose *pfeik1 *locus was disrupted. Clonal lines deriving from two independent transfection experiments were established by limiting dilution, and their genotypes were analysed by PCR (Figure 4B). The amplicon corresponding to the wild-type locus was not detected in clones C1 and C8 (lane 1), but was observed in wild-type parasites (lane 5). In contrast, PCR products that are diagnostic of both the 5' (lanes 3 & 7) and 3' (lanes 4 & 8) boundaries of the integrated plasmid were amplified from C8, but not 3D7 parasites (lanes 11 & 12). The C1 and C8 clones also yielded a signal with primers that are specific for the transfection plasmid, and detect retained episomes or integrated concatemers. Integration was verified by Southern blot analysis of *Hind*III-digested genomic DNA (Figures 4C and 4D); the 12 kb band containing the wild-type locus is replaced in clones C1 and C8 by the expected two bands (10.4 kb and 6.8 kb) resulting from integration. The remaining 5.3 kb band is derived from linearized plasmid, or from digestion of concatemers of plasmid (which may or may not be integrated into the chromosome). These results confirm that the *pfeik1 *locus was indeed disrupted in clones C1 and C8, and demonstrate PfeIK1 is not required for completion of the asexual cycle in *in vitro *cultures. Additionally, asexual parasite cultures were synchronized and carefully monitored through several life cycles; samples were taken every 30 minutes and assessed for DNA content by flow cytometry [42]. No significant difference was observed in asexual cycle duration of the parental 3D7 clone and that of *pfeik1*^- ^parasites; cycle times of 49.0 h +/- 0.5 and 49.2 h +/- 0.7, respectively, were measured (Figure 5).

### eIF2α is not phosphorylated in *pfeik1*^- ^clones during amino acid starvation

**Figure 6:**
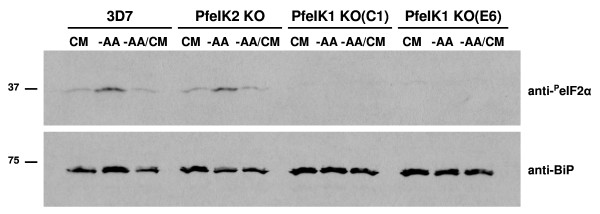
***pfeIK1*^- ^parasites do not phosphorylate eIF2α in amino acid-limiting conditions**. *pfeik1*^- ^clones C1 and E6, as well as the 3D7 parent clone and a *pfeik2*^- ^clone used as controls, were synchronized to the late ring stage and equally partitioned into individual cultures. Growth of the parasites was continued up to 5 hours at 37°C in either complete RPMI (CM) or in RPMI lacking amino acids (-AA). CM was added back to one amino acid-deprived culture, and re-incubated for an additional 45 minutes. Total lysates from the parasites were prepared for SDS-PAGE, followed by immunoblotting with antibodies against phosphorylated eIF2α (anti-phosho eIF2α). Antibodies against the endoplasmic reticulum marker BiP (anti-BiP) served as the loading control.

To determine whether *pfeik1*^- ^parasites were defective in responding to amino acid-limitation, we cultured these parasites in RPMI medium containing either all or no amino acids and assayed for eIF2α phosphorylation through western blot analysis (Figure 6). We observed that *pfeik1*^- ^parasites were unable to modulate the phosphorylation state of eIF2α in response to changing amino acid conditions, in direct contrast to wild-type parental clone 3D7. A further control was provided by performing the assay using a parasite clone lacking PfeIK2, another enzyme related to eIF2α kinases (see Figure 2A; a full characterisation of PfeIK2 and *pfeik2*^- ^parasite clones is to be published elsewhere). The *pfeik2*^- ^parasites, which were generated using the same strategy as that described here for *pfeik1 *and were therefore also resistant to blasticidin, readily phosphorylated eIF2α in amino acid starvation conditions, like wild-type 3D7 parasites. This demonstrates that the abolition of eIF2α phosphorylation observed in *pfeik1*^- ^parasites is not due to non-specific effects resulting from the genetic manipulations performed to obtain the mutant clones. Taken together, these data identify PfeIK1 as a crucial regulator of amino acid starvation stress response of intra-erythrocytic parasites.

### *pfeik1*^- ^clones are competent for sexual development and mosquito infection

**Figure 7:**
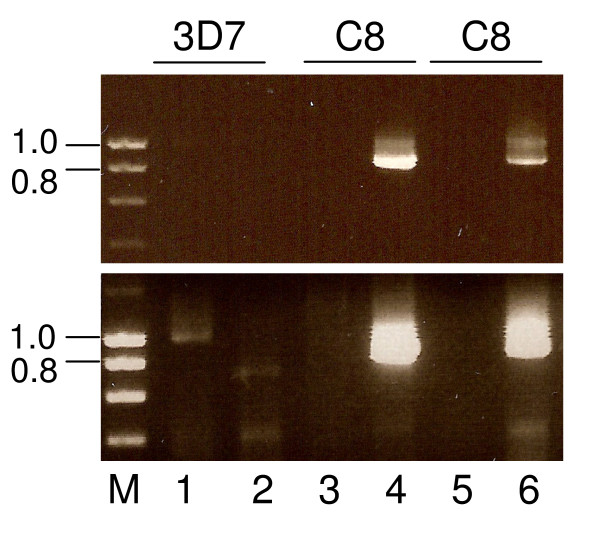
**Analysis of the parasite genotypes in mosquito infections**. Genomic DNA extracted from a wild-type 3D7-infected mosquito and from two mosquitoes infected with clone C8 was analysed by nested PCR; primer positions are indicated in Fig. 3A. The inner PCR product is shown. Lanes 1, 3 & 5 are diagnostic for the wild-type locus (primers 1 + 2, followed by 5 + 6). Lanes 2, 4 & 6 are diagnostic for the 3' boundary of plasmid integration (primers 3 + 2, followed by 7 + 6). The 3D7 infected mosquito used here serves as a control for PCR amplification of the wild-type locus from a midgut, but came from a separate experiment and hence did not provide a control for infection prevalence or intensity. Upper panel: shorter exposure; lower panel: longer exposure to reveal the wild-type band in lane 1 and its absence in the C8 samples. M = co-migrating markers.

The *pfeik1*^- ^parasites were able to differentiate into gametocytes (data not shown). Further, qualitative results showed that *pfeik1*^- ^male gametocytes were competent to differentiate into gametes (*in vitro *exflagellation). To investigate whether PfeIK1 plays an essential role in subsequent life cycle stages, mosquitoes were fed with cultures of *pfeik1*^- ^gametocytes. The numbers of oocysts associated with midguts dissected 10 days post-feeding, and the numbers of mosquitoes with sporozoite-positive salivary glands 16 days post-feeding, were then determined. This revealed that the complete sexual cycle can occur in the absence of PfeIK1, resulting in formation of oocysts and sporozoites (Table 1). Infection rates and median numbers of oocysts per infected mosquito are low relative to what is routinely observed in transmission experiments with the wild-type clone 3D7. However, this is to be expected from parasites that have been kept in continuous culture for a long period of time; in the present case it had taken ~7 months in culture to obtain knockout clones suitable for mosquito infection experiments. Circumstantial evidence that low infection levels are not a direct consequence of *pfeik1 *disruption is provided by the observation that our control for these experiments (mock-transfected 3D7 that had been cultured for the same duration, in parallel to the *pfeik1*^- ^parasites), had completely lost the ability to produce gametocytes and therefore infect mosquitoes. Importantly, to verify that the parasites infecting the mosquitoes had not reverted to the wild-type genotype, midguts from infected mosquitoes were collected 10 days post-feeding, from which total DNA was extracted and used in nested PCR experiments. The wild-type locus could be amplified from mosquitoes infected with wild-type 3D7 parasites, but not from those infected with *pfeik1*^- ^C8 parasites (Figure 7, lower panel, lanes 1, 3, 5). Conversely, the amplicon diagnostic of the 3' boundary of the integrated plasmid could only be amplified from midguts of *pfeik1*^- ^C8-infected mosquitoes, but not from mosquitoes infected with wild-type parasites (lanes 2, 4, 8).

**Table 1:** Oocyst and sporozoite formation by *pfeik1*^- ^parasite clones.

**Clone**	**Exp. no**	**% Infection (no. infected/no. dissected)**	**Median oocyst no. per infected mosquito (range)**	**% Sporozoite-positive (no. infected/no. dissected)**
C1	1	15% (2/14)	1.5 (1–2)	ND

C8	1	44% (7/16)	10 (1–34)	37% (7/19)

C8	2	20% (5/25)	2 (1–5)	ND

There was no difference in the prevalence of mosquitoes positive for the oocyst and sporozoites stages (exp. 2, p = 0.74).

On the basis of the similarities between PfeIK1 and GCN2, we hypothesized that PfeIK1 is involved in modulating the response to amino acid starvation depicted in Figure 1B. That this is indeed the case was demonstrated through a reverse genetics approach: parasites lacking PfeIK1 do not phosphorylate eIF2α in response to amino-acid depletion (Figure 6). Future work will determine the impact of PfeIK1 activation on both the rate of translation and the possible selection of specific messages that are translated under stress conditions. Overall, the data presented here suggest that eIF2α phosphorylation in response to amino-acid starvation is not essential to parasite survival during the erythrocytic asexual cycle (at least in an *in vitro *cultivation context), or for completion of sporogony.

Commitment to gametocytogenesis has been proposed to be linked to stress response, and eIF2α might possibly be involved in this process. At first sight, the data presented here suggest that PfeIK1 does not regulate gametocytogenesis, since *pfeik1*^- ^parasite are able to undergo sexual development. However, caution must be exercised, as compensatory mechanisms can be at play in knock-out parasites. Indeed, in a similar situation concerning another protein kinase family, it was observed that disruption of the gene encoding one of the two *P. falciparum *mitogen-activated protein kinases (MAPKs), *pfmap-1*, does not cause any detectable phenotype, but that *pfmap-1*^- ^parasites overexpress the second parasite MAPK, Pfmap-2 [40]. A similar compensation mechanism may operate between the three PfeIKs represented in the parasite kinome (Figure 2A). Even though compensatory mechanisms to permit sexual differentiation are presumably less likely to occur than those allowing the survival of asexual parasites (because of the absence of a true selection pressure), it cannot be formally excluded that PfeIK1 plays a role in gametocytogenesis in a wild-type parasite background. Investigating this possibility will require inducible and/or multiple knock-outs and the availability of mono-specific antibodies to monitor the levels of each PfeIK in parasites lacking one of them.

## Conclusion

Phylogenetic analysis indicates that the *P. falciparum *kinome includes three putative eIF2α kinases. One of these, PfPK4, was previously shown to phosphorylate a peptide corresponding to the target region of human eIF2α [27]. It is demonstrated here that PfeIK1 is able to phosphorylate the conserved regulatory site on the *Plasmodium *orthologue of the translation factor *in vitro*, and that eIF2α phopshorylation in response to amino-acid starvation does not occur in *pfeik1*^- ^parasites. The present study thus establishes that malaria parasites possess the molecular machinery that pertains to stress-dependent regulation of translation, and that this machinery is actually used in stress response.

## Competing interests

The authors declare that they have no competing interests.

## Authors' contributions

CF carried out molecular cloning, kinase assays, parasite genetic manipulations and analysis, participated in bioinformatic analysis and drafted the manuscript. SB carried out parasite starvation and immunoblotting experiments. IR analysed parasite growth. JW participated in sequence alignments and generated the phylogenetic tree. LRC carried out mosquito infections and participated in their analysis. DEG participated in conception of the study. CD conceived of the study, participated in its design and coordination and helped to draft the manuscript. All authors read and approved the final manuscript.
